# IL-1 Receptor Antagonist Anakinra Inhibits the Effect of IL-1β- Mediated Osteoclast Formation by Periodontal Ligament Fibroblasts

**DOI:** 10.3390/biology14030250

**Published:** 2025-02-28

**Authors:** Elizabeth Steemers, Wael M. I. Talbi, Jolanda M. A. Hogervorst, Ton Schoenmaker, Teun J. de Vries

**Affiliations:** 1 Department of Periodontology, Academic Centre for Dentistry Amsterdam, University of Amsterdam and Vrije Universiteit, Gustav Mahlerlaan 3004, 1081 LH Amsterdam, The Netherlands; elizasteemers@gmail.com (E.S.); w.talbi@acta.nl (W.M.I.T.); t.schoenmaker@acta.nl (T.S.); 2 Department of Oral Cell Biology, Academic Centre for Dentistry Amsterdam, University of Amsterdam and Vrije Universiteit, Gustav Mahlerlaan 3004, 1081 LH Amsterdam, The Netherlands; jma.hogervorst@acta.nl

**Keywords:** osteoclast, anakinra, periodontal ligament fibroblast, periodontitis, inflammatory bone disease

## Abstract

Rheumatoid arthritis and periodontitis are comorbidities that share mutual pathways. IL-1β is a pro-inflammatory cytokine that plays a crucial role in both diseases. One of the treatment options for rheumatoid arthritis is the use of an IL-1 receptor antagonist (IL-1RA) such as anakinra. Anakinra tempers the disease by decreasing bone resorption and it could possibly stimulate bone formation. Here, we investigate the effect of anakinra in a periodontal disease setting on osteoclastogenesis by co-culturing periodontal ligament fibroblasts (PDLFs) and peripheral blood mononuclear cells (PBMCs) that contain monocytes, a source of osteoclast precursors, as well as by culturing PBMCs alone. The effect of anakinra on PDLF-mediated osteogenesis was studied under mineralization conditions. To mimic a chronic infection such as that prevalent in periodontitis, 10 ng/mL of IL-1β was added either alone or with 10 µg/mL of anakinra. Osteoclastogenesis experiments were performed using co-cultures of PDLF and PBMCs and PBMCs only. Osteoclastogenesis was determined through the formation of multinucleated cells in co-cultures of PDLF and PBMCs, as well as PBMCs alone, at day 21, and gene expression through qPCR at day 14. Osteogenesis was determined by measuring alkaline phosphatase activity (ALP) per cell at day 14. Anakinra is effective in downregulating IL-1β mediated leukocyte clustering and osteoclastogenesis in the co-cultures of both PDLF and PMBCs and PBMCs alone. Gene expression analysis shows that IL-1β increases the expression of the osteoclastogenic marker RANKL and its own expression. This higher expression of IL-1β at the RNA level is reduced by anakinra. Moreover, IL-1β downregulates OPG expression, which is upregulated by anakinra. No effects of anakinra on osteogenesis were seen. Clinically, these findings suggest that anakinra could have a beneficial systemic effect on periodontal breakdown in rheumatoid arthritis patients taking anakinra.

## Simple Summary

Periodontitis is a disease of the gums with continuous inflammation that leads to loss of the bone in which the teeth are embedded. Such an inflammation is triggered by so-called inflammatory cytokines, which lead to the activation of the bone-eater cells or osteoclasts. Inhibition of such cytokines could be beneficial for the patient. This paper uses cells that are from the inflamed area, so-called periodontal ligament fibroblasts, to test whether the inhibitory molecule anakinra, which is clinically used in rheumatoid arthritis, another inflammatory bone disease of the joints that bears similarities with periodontitis, inhibits the formation of bone-eater cells. Thus, a cell model is used that mimics the microenvironment of the gums. These periodontal ligament fibroblasts can steer osteoclast formation. The results of this study show that when the inflammatory cytokine IL-1β is added, more osteoclasts form. However, when the inhibitor anakinra is added, this effect no longer occurs. At the level of inhibiting the bone-eating cell, the osteoclast, this study shows that medication that is useful for one disease, rheumatoid arthritis, and could be clinically interesting for the inflamed gums disease periodontitis, as well.

## 1. Introduction

Periodontitis is a complex, chronic inflammatory disease that causes destruction of the tooth-supporting apparatus and can lead to tooth loss [1]. Rheumatoid arthritis is a chronic, autoimmune, inflammatory systemic disease, which is characterized by persistent joint inflammation, and, like periodontitis, also can cause bone loss. There is growing evidence suggesting an association between periodontitis and rheumatoid arthritis [2,3]; in fact, there seems to be a bidirectional relationship. Patients with periodontitis have an increased risk of suffering from rheumatoid arthritis, and rheumatoid arthritis patients are at an increased risk of developing periodontitis [4]. Both diseases are chronic inflammatory diseases and share common pathobiological pathways. These include an inflamed microenvironment and serum cytokine concentration, matrix metalloproteinases, and other mediator profiles. An important hallmark of both diseases is furthermore osteoclast-mediated bone destruction [5]. Rheumatoid arthritis results in an increase in serological biomarkers such as ACPA, CRP, IL-1β, IL-6, and TNF-α [6]. These biomarkers are also elevated in the tissue of periodontitis patients [7] and are known to contribute to tissue destruction and bone resorption, for instance, by directly activating osteoclasts [8].

IL-1, both the cell-bound IL-1α and the secreted IL-1β, plays an important role in the pathogenesis of both rheumatoid arthritis and periodontitis. In rheumatoid arthritis patients, IL-1β induces prostaglandin E and collagenase production by synovial cells, promotes bone resorption, and upregulates the production of other pro-inflammatory cytokines, such as GM-CSF and IL-6 [9]. Likewise, in periodontitis patients, IL-1β triggers cell chemotaxis, collagen destruction via upregulating the secretion of matrix metalloproteinases (MMPs), and bone resorption by increasing osteoclastogenesis [10].

Considering that IL-1β is one of the signaling pathways of rheumatoid arthritis and periodontitis, a therapeutic option is the use of an IL-1 receptor antagonist (IL-1RA) that prevents IL-1β signaling. A safe and commonly used drug is anakinra. Anakinra blocks IL-1β and therefore stops pro-inflammatory signaling in general. It has an anti-inflammatory effect, as was demonstrated by a decrease in the CRP levels in rheumatoid arthritis patients [11]. Anakinra is the first approved IL-1β-targeted therapy. It is a glycosylated human recombinant IL-1RA, which binds to IL-1β and competitively prevents binding of both IL-1α and IL-1β with the IL-1 receptor 1 [12]. Inflamed pockets may contain citrullinated proteins. Pyroptosis may follow, giving rise to inflammasome activation, a process that is strongly associated with IL-1β release [13].

Several studies have shown that blocking the effects of IL-1β in rheumatoid arthritis protects bone and cartilage [14]. In vitro studies on cartilage tissue have shown that IL-1RA reduced the synovial fibroblast-mediated destruction by up to 45% [15]. In vitro studies on bone slices have shown that IL-1RA reduced the amount of bone resorption and the ability to block formation of osteoclast-like cells when cultured in the presence of IL-1β [16,17,18]. Animal studies showed that IL-1RA reduced cartilage destruction and bone erosion [19,20]. Furthermore, IL-1RA knock-out mice have an increased number of osteoclast precursors, in particular in the jaw and long bone [21]. Depending on the type of osteoclast precursor, IL-1β, as activator of osteoclasts, affects the activity and life-span of osteoclasts [22]. In a clinical trial it was observed that IL-1RA reduced radiologic progression of rheumatoid arthritis [23], conceivably by inhibiting IL-1’s effect on osteoclast activity. Anakinra is therefore a safe and effective drug used to treat rheumatoid arthritis [24].

Although the effect of IL-1RA on osteoclasts is more widely discussed in the literature, studies regarding the effect on osteoblasts are scarcer. The study by Guo et al. [25] shows that IL-1α induces the apoptosis of osteoblasts and inhibits osteoblast differentiation. We can therefore hypothesize that an IL-1RA would counteract this effect, resulting in more active osteoblasts.

Given the parallel between rheumatoid arthritis and periodontitis, it is remarkable that anakinra, as a potent inhibitor of the common denominator IL-1β, has not been studied in models for periodontitis. These interesting findings from clinical, in vitro, and in vivo studies support the need for further research using a relevant cell system. PDL fibroblasts (PDLFs) are connective tissue cells that anchor teeth in bone and play a key role in both osteoclastogenesis and osteogenesis. Both of these processes can be mimicked in vitro. PDLFs have a dual role in both inhibiting and promoting osteoclastogenesis. Under physiological conditions, PDLFs produce higher levels of OPG than RANKL, thereby inhibiting osteoclastogenesis. PDLFs have the capacity to attract osteoclast precursors and enable their migration to the bone surface. The fusion of mononuclear precursors of the monocyte lineage gives rise to osteoclasts. Such differentiation is called osteoclastogenesis. In the context of IL-1β, PLDFs have been pre-treated with IL-1β for 6 h and were shown to have a long-lasting effect on the adherence of osteoclast precursors, subsequent osteoclast formation, and even IL-1β expression [26]. These culture conditions probably do not mimic chronic conditions where long-lasting IL-1β signaling could be considered. Another source of IL-1 signaling could come from PBMCs only, which make IL-1 and may use this for their spontaneous formation of osteoclast-like cells at high density [27,28,29,30,31]. For this study, we therefore studied both PDLF-PBMC co-cultures and PBMC monoculture osteoclastogenesis assays in the presence of IL-1β and its clinically used inhibitor anakinra.

The aim of this in vitro study was to assess the IL-1β inhibitory effect of anakinra on osteoclastogenesis and osteogenesis. We hypothesized that anakinra has an inhibitory effect on osteoclastogenesis, both in the absence and in the presence of IL-1β.

## 2. Materials and Methods

### 2.1. Study Design and Cell Cultures

This in vitro study followed the EQUATOR guidelines for such an in vitro study. Ethical approval was obtained by the ethical review committee (ETC) of the Academic Centre of Dentistry Amsterdam (ACTA) under the number ETC ID: 2021-36394.

PDLFs were retrieved from extracted wisdom teeth from patients in their early–late teens or early twenties (17–22 years old). Cells were scraped off the middle one-third of the root, chopped into pieces, and added to 6-well plates containing DMEM (Gibco BRL, Paisley, UK), 10% fetal clone I FCI (Hyclone, Logan, UT, USA), and 1% of the antibiotics penicillin, streptomycin, and fungizone (PSF, Sigma-Aldrich, Saint Louis, MO, USA). Upon confluent outgrowth, cells were trypsinized and transferred to a 75 cm^2^ flask (passage 1). When confluence was reached, the cells were transferred to two 175 cm^2^ flasks (passage 2). When confluence was reached here, cells were frozen in 6 aliquots containing the cells in 90% FCI and 10% DMSO (passage 3) and placed in liquid nitrogen. Previously, we showed that these cells contain the characteristic gene expression of PDLFs, including periodontal ligament associated protein PLAP-1 and fibroblast associated protein-α FAP-α [31]. Cells from passage 5 were used in all experiments. PDLFs were anonymized immediately after extraction for research purposes, according to Dutch law. PBMCs were derived from buffy coats from the blood bank (Sanquin, Amsterdam, The Netherlands). The researchers could not trace the identity of the donors.

The first experiment consisted of a titration in order to determine the effect of anakinra on osteoclastogenesis using different concentrations of anakinra (0, 0.01, 0.1, 1, and 10 μg/mL). Osteoclastogenesis experiments were performed using co-cultures of PDLFs and PBMCs, as well as PBMCs only. Osteogenesis was determined through alkaline phosphatase activity and DNA measurements. Since no IL-1β was added in the titration experiment, this experiment assessed the role of anakinra in blocking possible endogenous activity of IL-1β. Results were based on 3 PDLF donors as biological replicates and on a quadruplicate plating of PBMCs when plated without PDLFs.

In order to further study the effectiveness of anakinra in blocking IL-1β (Biotechne, Minneapolis, MN, USA), these experiments were followed-up with experiments where 10 ng/mL IL-1β was added together with 10 µg/mL of anakinra that followed from the titration experiment. Outcomes of this experiment were based on seven PDLF donors, or in the case of PBMC alone, on a quadruplicate plating. Several techniques were used to assess the effect of anakinra on osteoclastogenesis. Firstly, it became apparent that leukocyte clusters formed when IL-1β was added. The cluster formation was analyzed at day 14. Secondly, the formation of multinucleated tartrate resistant acid phosphatase (TRAcP)-positive cells was scored as an outcome of osteoclastogenesis, both in co-cultures of PDLF and PBMCs, and in PBMC-only cultures at day 21. Thirdly, gene expression analysis through quantitative polymerase chain reaction (qPCR) was performed at day 14. The effect of anakinra and its inhibition on osteogenesis was assessed by measuring alkaline phosphatase activity.

### 2.2. Osteogenesis and Osteoclastogenesis Assays Using PDLF

**Figure 1:**
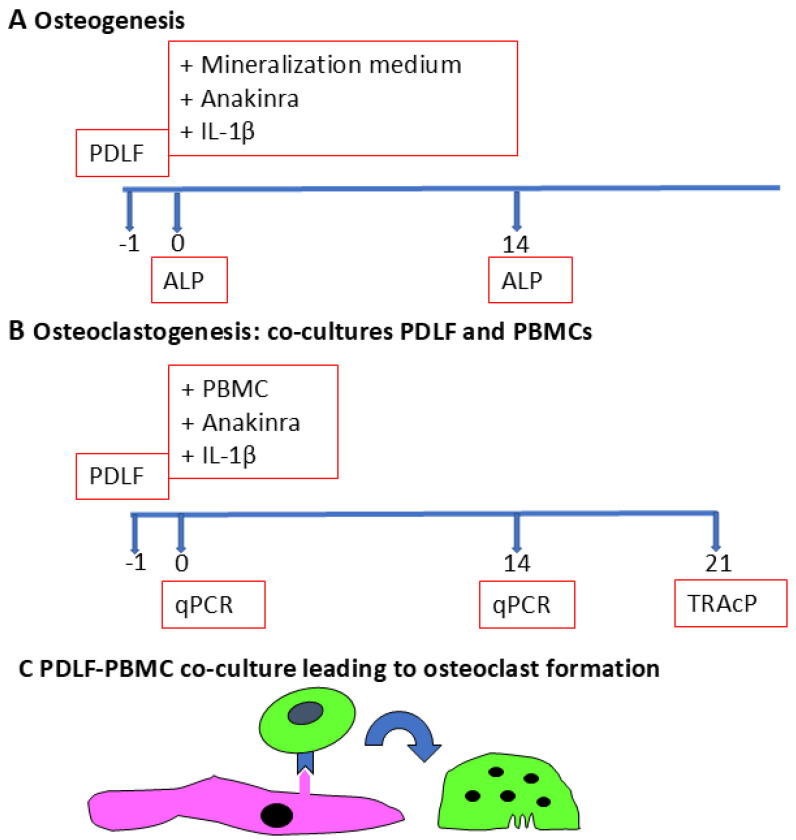
Outline of experiments. Timeline in days. The periodontal ligament fibroblasts (PDLFs) were seeded 1 day before the start of the experiment. Anakinra was added to the osteogenesis (**A**) and osteoclastogenesis (**B**) experiments. (**C**) A graphical explanation of the co-culture osteoclastogenesis experiments, with the PDLFs in pink and the osteoclast precursor that differentiates into a multinucleated osteoclast in green.

All experiments with PDLFs were performed with cells from the 5th passage, using 48-well plates. For the osteoclastogenesis experiments, PDLFs were seeded at 1.5 × 10^4^ cells per well; for the osteogenesis experiments, 3 × 10^4^ cells per well were used (day −1, see Figure 1). The culture medium was removed from the 48-well plates at the start of the experiment (day 0). In the osteogenesis-assay plates, cells were cultured in 0.4 mL of normal medium, which consisted of DMEM, 10% FCI (HyClone, Logan, UT, USA), and 1% penicillin, streptomycin, and amphotericin (PSF, Sigma, St. Louis, MO, USA), or in mineralization medium that contained extra 50 μg/mL ascorbic acid (Sigma) and 10 nM β-Glycerophosphate (Sigma), and various concentrations of anakinra (for the 0 mM anakinra condition, the solvent volume of sterile water was used). Anakinra, or the clinical trade name Kineret (Sobi, Waltham, MA, USA), was a generous gift from Dr. Sietse Q. Nagelkerke, pediatric rheumatologist, Sanquin, the Netherlands. PBMCs from a buffy coat (Sanquin, Amsterdam, The Netherlands) were added for the osteoclastogenesis assays. For the first experiment, different concentrations were used, namely 0, 0.01, 0.1, 1, and 10 μg/mL.

After the titration experiment, the following conditions were applied: 1. Control condition without any additions, 2. IL-1β 10 ng/mL (Biotechne, Minneapolis, MN, USA), 3. a combination of 10 ng/mL IL-1β and 10 µg/mL anakinra, or 4. 10 µg/mL anakinra alone. In the course of 21 days of the experiment, the culture and mineralization medium were replenished twice a week.

### 2.3. DNA Concentration and Alkaline Phosphatase Activity

Cells were harvested at days 0 and 14 of culturing. Cells were lysed in 200 μL MilliQ per well and stored at −20 °C until analysis. Plates underwent three cycles of freezing and thawing. They were then transferred to Eppendorf reaction tubes after standardized removal by scraping with the pipette tip from the bottom of the well. Afterwards, centrifugation was performed for 10 min. The samples were pipetted in duplicate and then incubated at 37 °C for one hour. Alkaline phosphatase (ALP) activity of the cell lysate was measured using 4-nitrophenyl phosphate disodium salt (Merck, Darmstadt, Germany) at pH 10.3 as a substrate for ALP according to the method described by Prins et al. [32]. After incubation of 60 min at 37 °C, the reaction was stopped with sodium hydroxide. Absorbance was measured at 405 nm with a Synergy HT spectrophotometer (BioTek Instruments Inc., Winooski, VT, USA). DNA concentration (ng/mL) was measured using a CyQuant Cell Proliferation Assay Kit (Molecular Probes, Leiden, The Netherlands). Fluorescence was read at 485 nm excitation and 528 nm emission with a Synergy HT spectrophotometric microplate reader (Agilent technologies, Santa Clara, CA, USA). ALP activity was expressed as ALP per DNA (nmol/ng DNA).

### 2.4. Quantitative Polymerase Chain Reaction

Quantitative polymerase chain reaction (qPCR) analysis was performed for osteoclastogenesis at day 0, before the addition of PBMCs, and at day 14. At these time points, the culture medium was removed and RNA lysis buffer (Qiagen, Hilden, Germany) was added per well. Subsequently, the 48-well plates were stored at −80 °C until further use. RNA isolation was performed with a Qiagen RNeasy Mini kit according to the manufacturer’s instructions. The RNA concentration and quality were determined using absorption read at 260 and 280 nm with a Synergy HT spectrophotometer (BioTek Instruments Inc., Winooski, VT, USA). RNA was reverse transcribed to cDNA with the MBI Fermentas cDNA synthesis Kit (Vilnius, Lithuania). Oligo(dT) 18 and D(N)6 were used as primers. Real-time primers were designed for several genes (Table 1). PCR was performed on the LC480 light cycler (Roche, Basel, Switzerland). Hypoxanthine phosphoribosyltransferase 1 (HPRT1) was used as the housekeeping gene for the osteoclastogenesis and osteoclast markers. The included osteoclastogenesis markers were RANKL, OPG, RANKL/OPG, IL-1β, IL-1RA, TNF-α, DC-STAMP, and TRAcP. Gene expression was normalized for HPRT1 expression following the comparative threshold (Ct) method. ΔCt (Ct gene of interest—Ct housekeeping gene) was calculated and relative expression of the genes was determined as 2^−ΔCt^.

### 2.5. TRAcP Staining and Osteoclast Quantification

Osteoclast quantification was performed after 21 days of culturing for both experiments. Cells were washed with PBS and fixed with 4% PBS buffered formaldehyde for 10 min before being stored with PBS at 4 °C. After washing the cells with water at 37 °C, TRAcP staining solution was made, which is made up of Fast Garnet GBC base, sodium nitrate, acetate, naphthol AS-BI, and tartrate in AD at 37 °C, using the leukocyte acid phosphate kit (Sigma) following the manufacturer’s instructions. The nuclei were then counterstained with DAPI (diamidino-2-phenylindole dihydrochloride). Counting of multinucleated osteoclasts (at least 3 nuclei) was performed. For the final experiment, a distinction was made between cells with 3–5 nuclei, and cells with ≥6 nuclei. For analyses, the average of the number counted in the duplicate wells per donor was used.

### 2.6. Statistical Analysis

The effects on the cells of the different conditions containing IL-1β and anakinra were compared using one-way ANOVA followed by the non-parametric Friedman test, using GraphPad Prism software, version 12. Dunn’s multiple comparison test was conducted as a post-test. A *p*-value of <0.05 was the cut-off for statistical significance.

## 3. Results

The first experiments established the effect of various concentrations of anakinra both on osteoclast formation and on osteogenesis. These experiments were performed to test inhibition of possible endogenously produced IL-1α and IL-1β.

**Increased concentration of anakinra inhibits osteoclast formation in PBMC cultures**

**Figure 2:**
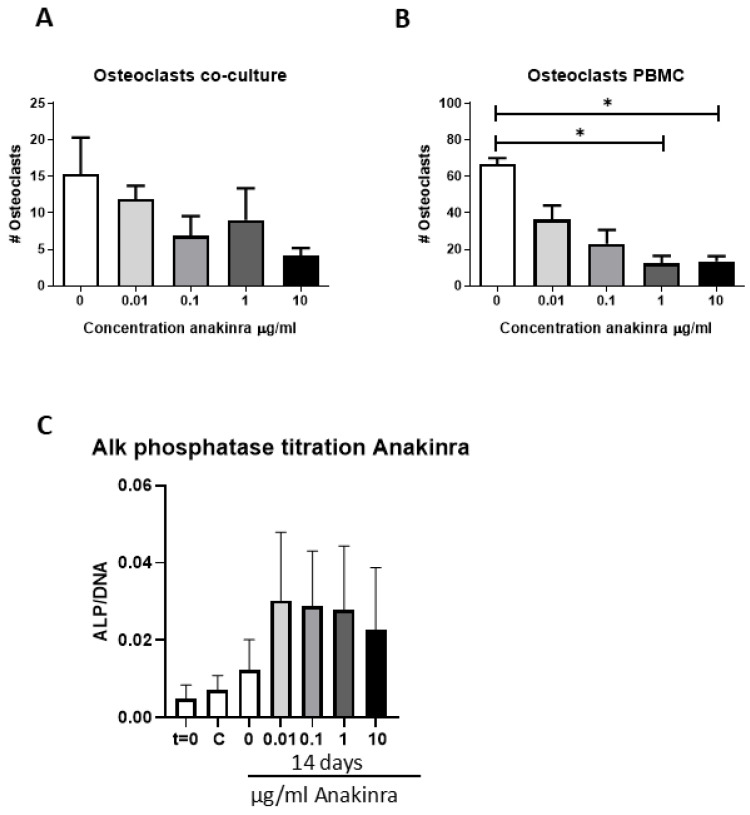
(**A**–**C**): Titration experiments with anakinra on osteoclast formation (**A**,**B**) and alkaline phosphatase activity in osteogenesis experiments (**C**). (**A**) Number of osteoclasts in co-culture of PDLF and PBMCs. (**B**) Number of osteoclasts in PBMC-only culture. For these experiments, 3 PDLF donors were used in (**A**). PBMCs only, in (**B**), were seeded in quadruplicate. *: *p* < 0.05. (**C**) Increased concentration of anakinra does not affect alkaline phosphatase activity. ALP/DNA (nMol/ng DNA): alkaline phosphatase enzyme activity per cell at days 0 (**C**) and 14 (C—normal medium without anakinra, 0.01, 0.1, 1, 10 μg/mL anakinra in mineralization medium). Activity was measured at day 0 (t = 0) and at day 14 for all subsequent measures. C = control without mineralization medium, M = with mineralization medium. Results from 3 PDLF donors.

The effect of anakinra was established both in co-cultures of PDLF and PBMCs using three PDLF donors. Though a clear trend was observed, no significant effect of anakinra concentrations was observed in co-cultures (Figure 2A). A second osteoclastogenesis assay, using a high density of PBMC, showed that the number of osteoclasts significantly decreased at the concentrations 1 μg/mL and 10 μg/mL compared to 0 μg/mL. Moreover, the effect seemed to be dose-dependent as the number of osteoclasts decreased as the concentration of anakinra increased from 0.01 to 10 μg/mL (Figure 2B). Based on these findings, the concentration of 10 μg/mL anakinra was used for further experiments.

**Increased dosage of anakinra does not affect osteogenesis**

Three PDLF donors were used to establish the effect of anakinra on osteogenesis. As a proxy for osteogenesis, ALP activity was measured and compared between the control group, mineralization group, and the different concentrations of anakinra with mineralization medium. Compared to day 0, there seemed to be an increase in ALP activity per cell at 14 days, but there was no difference between the various concentrations of anakinra (Figure 2C). Based on these results, 10 μg/mL was used for further experiments.

The goal of these initial titration experiments was to establish the effect and also the tolerance of anakinra and its possibly inhibitory effect on endogenously produced IL-1Β. To further mimic a chronically inflamed environment such as that apparent in periodontitis, we added 10 ng/mL IL-1β to osteogenesis and osteoclastogenesis cultures for the duration of the experiment. These experiments will then elucidate the effect of IL-1β on cellular processes, as well as whether anakinra can modulate these processes. For these experiments, seven PDLF donors were used.

**IL-1β induces large osteoclasts in high-density PBMC cultures, anakinra inhibits this effect**

**Figure 3:**
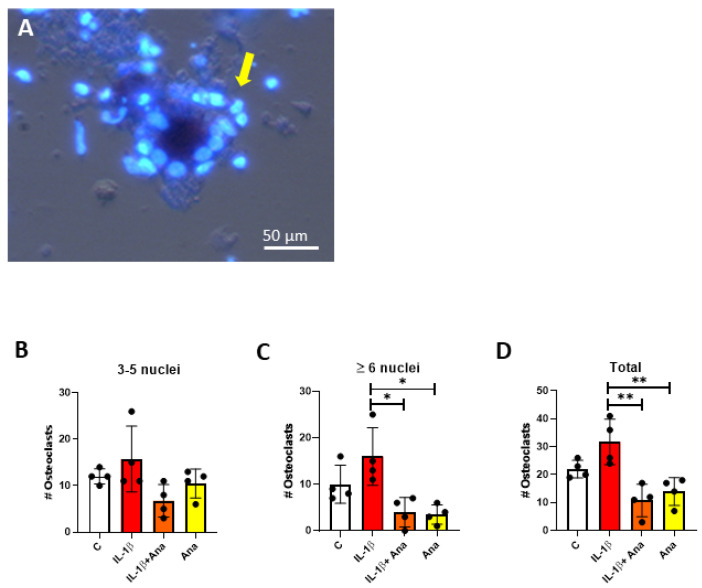
(**A**–**D**): IL-1Β induces large osteoclasts and this is reduced with anakinra in cultures containing only PBMC at high density. PBMCs were cultured and osteoclast formation was assessed at 21 days after TRAcP staining. (**A**) Yellow arrow: osteoclast with more than 6 nuclei. (**B**) Number of osteoclasts with 3–5 nuclei in PBMC culture. (**C**) Osteoclast count with ≥6 nuclei in PBMC culture. (**D**) Total osteoclast nuclei (≥3 nuclei). *: *p* < 0.05, ** *p* <0.01. Results are from quadruplicate plating of PBMCs.

Using the high-density cultures containing only PBMC, it was found that the number of osteoclasts with ≥6 nuclei was significantly increased when IL-1β was added (Figure 3A–D). Addition of anakinra in combination with IL-1β leveled the number of osteoclasts to control levels, most strikingly for the larger osteoclasts (Figure 3C). The IL-1β effect and the leveling by anakinra was not significant for smaller osteoclasts (Figure 3A), but for larger osteoclasts (Figure 3C) and the total number of osteoclasts (Figure 3D). These results indicate that chronic exposure of IL-1β increases the number of (large) osteoclasts and anakinra can nullify this effect.

**IL-1β induced leukocyte clusters disappear through anakinra in co-cultures of PDLF and PBMC**

**Figure 4:**
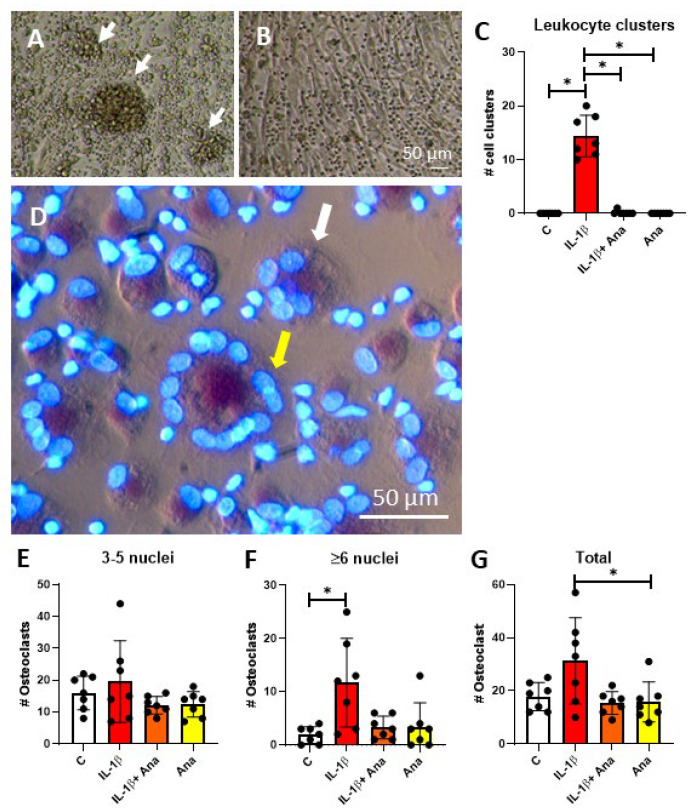
(**A**-**C**): IL-1Β induces cluster formation and subsequent osteoclast formation in co-cultures of PDLF and PBMCs. (**A**) Cluster formation (white arrows) in the presence of IL-1Β at 14 days. (**B**) Absence of clusters in the case of IL-1Β and anakinra (Ana). (**C**) Number of leukocyte cell clusters under the four conditions (control, IL-1Β, IL-1Β with anakinra, anakinra). (**D**–**G**): IL-1Β induces more osteoclasts with a higher number of nuclei in PDLF-PBMC co-cultures at day 21. Anakinra decreases the total osteoclast count at day 21. (**D**) Yellow arrows: osteoclasts with more than 6 nuclei; white arrows: osteoclast with less than 6 nuclei. (**E**) Number of osteoclasts with 3-5 nuclei in PDLF-PBMC co-culture. (**F**) Number of osteoclasts with ≥6 nuclei in PDLF-PBMC co-culture. (**G**) Total osteoclasts ≥3 nuclei in PDLF-PBMC co-culture. *: *p* < 0.05. Experiments from 7 PDLF donors are shown.

A very striking observation during co-culture of PDLF and PBMC was the appearance of leukocyte clusters when IL-1β was added. Previously, we showed that such leukocyte clusters correlate with the ultimate formation of osteoclasts [33,34]. IL-1β induced clusters at day 14 (Figure 4A), which were absent when anakinra was added (Figure 4B), as well as in all other conditions (Figure 4C). These data indicate that IL-1β induces leukocyte clusters, here in the presence of PDLF, and that anakinra is able to inhibit this formation.

**IL-1β induces more osteoclasts in PDLF-PBMC co-cultures**

Next, the number of osteoclasts after 21 days of co-culture was assessed, using TRAcP staining for the cytoplasm and DAPI staining for the nuclei to assist the visualization (Figure 4D). There were no significant differences between the conditions for the number of smaller osteoclasts with 3–5 nuclei (Figure 4E). However, larger osteoclasts with ≥6 nuclei (Figure 4F) were significantly more abundant in the IL-1β-treated group compared to the control group. In addition, the number of osteoclasts was comparable between the anakinra-treated groups and the control group. However, these results were not statistically significant compared to the IL-1β-treated group. In the analysis of the total number of osteoclasts, the number of osteoclasts in the anakinra group was significantly lower than that in the IL-1β group (Figure 4G).

**Anakinra nullifies the IL-1β effects on gene expression of osteoclastogenesis genes RANKL and OPG**

**Figure 5:**
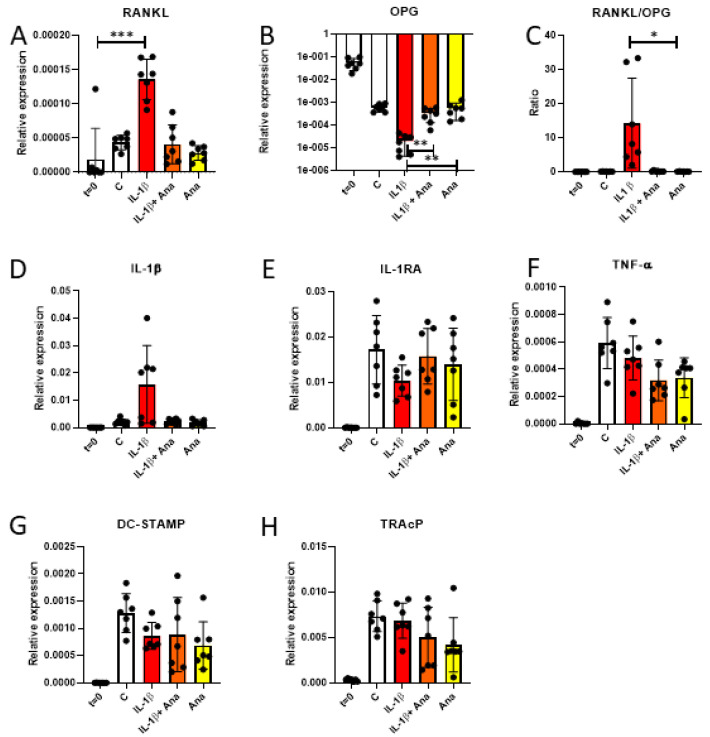
(**A**–**H**): Gene expression at 0 days or 14 days (PDLF-PBMC co-cultures without addition (**C**) or with IL-1Β, IL-1Β with anakinra and anakinra) of RANKL (**A**), OPG (**B**), ratio RANKL/OPG (**C**), IL-1Ββ (**D**), IL-1ΒRa (**E**), TNF-α (**F**), DC-STAMP (**G**), TRAcP (**H**). All data are from 7 PDLF donors. RANKL was increased by IL-1Β. Anakinra increased the expression of OPG. The ratio RANKL/OPG reduced in the anakinra group. *: *p* < 0.05, **: *p* < 0.01, ***: *p* < 0.001.

In search for an explanation for the IL-1β-mediated increased formation of larger osteoclasts, gene expression was assessed at t = 0 days and 14 days. RANKL expression was significantly increased in the IL-1β-treated group at t = 14 compared to t = 0 (Figure 5A). Furthermore, compared to the IL-1β-treated group, OPG expression was higher when anakinra was added (Figure 5B). The subsequent RANKL/OPG ratio was significantly higher in the IL-1β group compared to the anakinra group (Figure 5C).

**IL-1β nor anakinra affects IL-1β, IL-1RA or TNF-α expression**

Expression of inflammatory cytokines can directly stimulate osteoclasts. Also, inhibition of IL-1β could possibly affect expression of TNF-α as a compensatory mechanism. IL-1β, IL-1RA, and TNF-α were assessed in co-cultures at day 0 and day 14. Although no differences were observed (Figure 5D–F), the effect of IL-1β treatment on IL-1β expression was clearly heterogeneous: some co-cultures responded, others did not.

**IL-1β nor anakinra affected gene expression of osteoclast markers TRAcP and DC-STAMP**

Osteoclast-related genes TRAcP and DC-STAMP were not expressed by the PDLF at 0 days, the time point before osteoclast precursors containing PBMCs were added. At 14 days, no significant differences were observed between the culture conditions for expression of TRAcP (Figure 5G) or DC-STAMP (Figure 5H).

**Osteogenesis is not influenced by IL-1β or anakinra**

**Figure 6:**
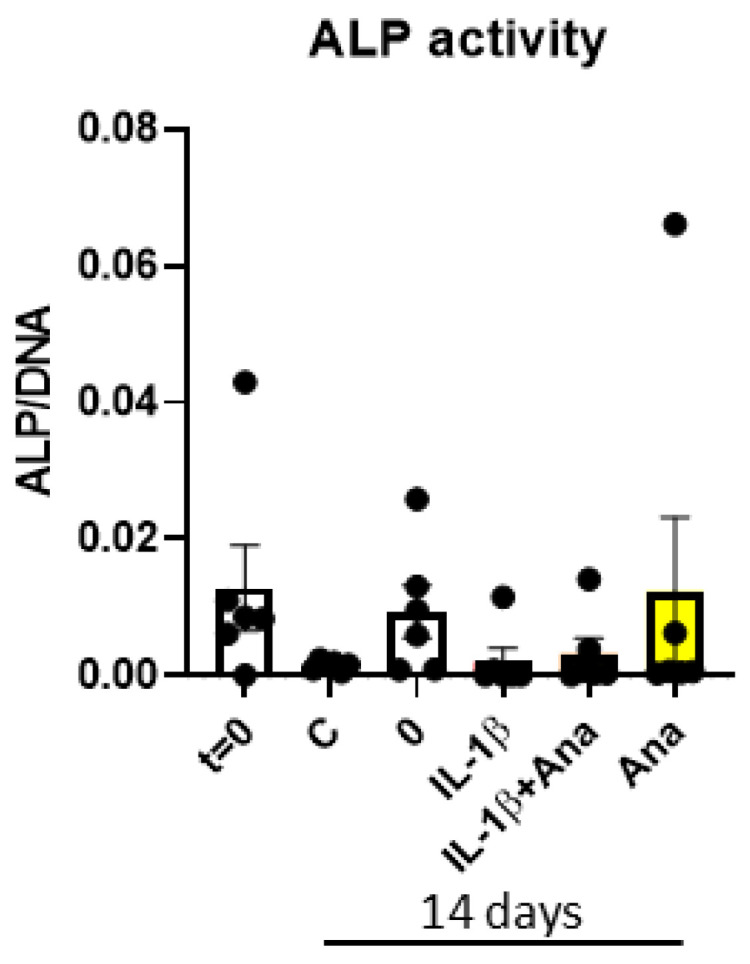
IL-1β or anakinra does not affect alkaline phosphatase activity. ALP/DNA in nMol/ngDNA: alkaline phosphatase enzyme activity per cell at day 0 (C) and 14 (C—normal medium without anakinra, last 4 columns, 0, IL-1β, IL-1β+Ana and Ana display the result where mineralization medium was added. Results from 7 PDLF donors are shown.

Alkaline phosphatase as a proxy for osteogenesis was measured at 0 and 14 days. No statistically significant differences were found in ALP activity between the four groups, indicating no significant differences in osteogenesis among them. Large variation was observed between the donors, indicating heterogeneity between the donors (Figure 6).

## 4. Discussion

The present study is the first to investigate the effect of the clinically used IL-1β inhibitor anakinra in assays investigating osteogenesis and osteoclastogenesis, in the context of periodontal ligament fibroblasts. The role of IL-1β in promoting bone resorption by increasing osteoclastogenesis has been highlighted in various studies [9,10]. Moreover, the effect of IL-1RA in reducing bone resorption and osteoclastogenesis has been demonstrated [15,19,23]. The effect of IL-1RA on osteogenesis has been less investigated but nevertheless seem to indicate a possible beneficial effect on osteogenesis in studies using the mouse osteoblasts MC3T3-E1 [25] or human stem cells [35].

Our original hypothesis regarding osteogenesis was that anakinra could possibly stimulate osteogenesis, since blocking endogenous or added IL-1β is anti-catabolic. This hypothesis has to be rejected. The addition of anakinra did not result in a statistically significant increase in ALP activity, nor in the first, titration experiment with anakinra, nor in the experiment using anakinra in the presence or absence of IL-1β.

Anakinra inhibited osteoclastogenesis in the PBMC culture, as seen by the reduction in osteoclast counts. This reduction was dose-dependent. A similar result was seen in the PDLF and PBMCs co-culture, although it was not statistically significant. Based on these results a concentration of 10 μg/mL of anakinra was used for the subsequent experiments. Usually, cytokines are expressed in the pg/mL or ng/mL range; therefore, such a 1000-fold excess concentration should be able to inhibit the added 10 ng/mL IL-1β or the endogenously produced IL-1β. IL-1β in the further experiments using PBMC culture, the addition of anakinra to IL-1β, as well as anakinra alone, decreased the number of osteoclasts with more than six nuclei as well as the total number of osteoclasts. Regarding the number of osteoclasts with 3–5 nuclei, there were no statistical differences. It therefore seems that the effect of anakinra is more visible in osteoclasts with a larger number of nuclei, indicating a role for IL-1β in the formation of especially larger osteoclasts. This could be specific for the two systems used here: the PBMC-only cultures and the co-cultures with periodontal ligament fibroblasts.

In addition, IL-1β induced cluster formation, as was visible by a spike in cluster formation at day 14 when IL-1β was added, especially in the co-cultures. In contrast to the PBMC-only cultures, the co-cultures include a cell type, the periodontal ligament fibroblast, that is relevant for the study of periodontal disease. In this context, these clusters were not visible when anakinra was added, indicating that anakinra blocked this IL-1β effect. This shows that that the formation of leukocyte aggregation is an exclusive characteristic feature of IL-1β. Such cell cluster formation was also seen in the study of de Vries and others [33] and was abolished when TNF-α infliximab was added. Also, in the study of Oostlander et al. [34], which investigated osteoclastogenesis in patients with Crohn’s disease, clusters were apparent in Crohn’s disease cultures. Both these studies could correlate the formation of leukocyte clusters with the formation of osteoclasts.

The osteoclastogenesis results of the co-cultures of PDLF and PBMCs showed more large osteoclasts with six and more nuclei when IL-1β was added. Importantly, anakinra could block this effect, indicating that it is an active inhibitor. This is in line with studies showing a decrease in the formation of osteoclast-like cells when IL-1RA was added [16,17,18]. We are the first to show this effect in a periodontitis model using periodontal ligament fibroblasts.

The effect of anakinra on osteoclastogenesis was demonstrated further with the typical osteoclastogenesis genes RANKL and OPG and the ratio between them. Firstly, IL-1Ββ increased the levels of RANKL gene expression and, secondly, anakinra decreases the gene expression of the ratio RANKL/OPG. The upregulation of RANKL by IL-1Β was also seen in an in vivo study in mice [36]. The downregulation of the RANKL/OPG ratio in these co-cultures shows that anakinra affects this ratio and subsequent osteoclast formation. This is in line with what has been described in the review by Ruscitti [37], which showed that several studies have shown that IL-1β increases RANKL and RANKL/OPG, thereby leading to bone resorption. Novel here is that we demonstrated that anakinra can modulate the IL-1β effect in the context of periodontal ligament fibroblast-mediated osteoclastogenesis.

Although the expression of IL-1β was not assessed at the protein level, it is expression at the RNA level was assessed. The expression of IL-1β, IL-1RA, TNF-α, DC-STAMP, and TRAcP did not statistically differ between the groups. It is important to note that there is certain heterogeneity between the donors, which therefore influences statistical testing. For instance, the levels of IL-1β gene expression were much higher in the IL-1β group, although this result was not statistically significant. The dots in the graph clearly show a dichotomy: some of the donors responded to IL-1β by increased IL-1β expression, whereas others did not respond. A shortcoming in our study is that we only assessed one timepoint. Bloemen et al. [26] have previously demonstrated that an incubation for 6 h with IL-1β caused increased expression of IL-1Ββ when assessed after 3 days of co-culture. TNF-α is also a pro-inflammatory cytokine, considered to have functions overlapping with those of IL-1β; for instance, they both cause an increase in osteoclast formation [22]. One could speculate that blockade of IL-1β signaling by anakinra could result in increased expression of TNF-α. However, this seems not to be the case, as TNF-α expression was not influenced. DC-STAMP is involved in cell fusion between osteoclast precursors during osteoclast development. In our study, it shows that neither IL-1β nor anakinra affects this expression. If fusion is initiated earlier than 14 days, earlier time points should have been assessed for DC-STAMP.

## 5. Conclusions

Based on the findings of this study, we can conclude that IL-1β, when added throughout the culture period as a mimic of a chronic inflammation such as periodontitis, induces osteoclastogenesis. The clinically relevant inhibitor of IL-1β, anakinra, then clearly downregulates this process. The effect of anakinra on osteoclastogenesis was proven through cell cultures of PDLF and PBMCs and PBMCs alone, as well as through the expression of osteoclastogenic gene markers. Using periodontal ligament fibroblasts, there seems to be no effect of anakinra on osteogenesis, suggesting its effect is directly on the osteoclast precursors rather than via the fibroblasts. This is the first in vitro study examining the effect of anakinra on osteoclastogenesis using a cell culture design of PDLF and PBMCs and PBMCs alone. Based on these studies, one could consider studying, in a cohort of rheumatoid arthritis patients, whether treatment with anakinra improves the periodontal status of these patients. For anti-TNF medication, this has been well studied, where anti-TNF medication improved the periodontal parameters plaque index, gingiva index, bleeding on probing, and probing pocket depth [38]. For the use of anakinra, which is used less abundantly and has also not been used as long as anti-TNF, such data do not exist, so it is too premature to state that the use of anakinra is to the benefit of both. On the other hand, PD is an infection-induced inflammatory disease. Blocking IL-1β might reduce the inflammatory burden, but might also impair immune response, allowing increased growth of the oral bacteria. This would once again demonstrate that treating patients with anti-inflammatory biologicals could benefit the status of a comorbidity, here periodontitis, for the benefit of both diseases.

## Figures and Tables

**Table 1:** Primer sequences of quantitative real-time PCR analysis.

Gene	Primer Sequence (5′ to 3′)	Amplicon Length (bp)	Ensembl Gene ID
β2-microglobulin	Forward: AAGATTCAGGTTTACTCACGTC Reverse: TGATGCTGCTTACATGTCTCG	293	ENSG00000166710
RANKL	Forward: CATCCCATCTGGTTCCCATAA Reverse: GCCCAACCCCGATCATG	60	ENSG00000120659
OPG	Forward: CTGCGCGCTCGTGTTTC Reverse: ACAGCTGATGAGAGGTTTCTTCGT	100	ENSG00000164761
DC-STAMP	Forward: ATTTTCTCAGTGAGCAAGCAGTTTC Reverse: AGAATCATGGATAATATCTTGAGTTCCTT	101	ENSG0000016493
TRAcP	Forward: CACAATCTGCAGTACCTGCAAGAT Reverse: CCCATAGTGGAAGCGCAGATA	128	ENSG00000102575
IL-1β	Forward: CTTTGAAGCTGATGGCCCTAAA Reverse: AGTGGTGGTCGGAGATTCGT	100	ENSG00000125538
IL-1RA	Forward: GCTGGATACTTGCAAGGACCAA Reverse: ACTCGTCCTCCTGGAAGTAGA	364	ENSG00000136689
TNF-α	Forward: CCCAGGGACCTCTCTCTAATCA Reverse: TGAGGGTTTGCTACAACATG	103	ENSG00000111956

## Data Availability

Data will be shared upon request.

## References

[1] Loos B.G., Van Dyke T.E. (2020). The role of inflammation and genetics in periodontal disease. Periodontology 2000.

[2] Berthelot J.M., Le Goff B. (2010). Rheumatoid arthritis and periodontal disease. Jt. Bone Spine.

[3] Rutger Persson G. (2012). Rheumatoid arthritis and periodontitis-inflammatory and infectious connections. Review of the literature. J. Oral Microbiol..

[4] de Pablo P., Dietrich T., McAlindon T.E. (2008). Association of periodontal disease and tooth loss with rheumatoid arthritis in the US population. J. Rheumatol..

[5] Bingham C.O., Moni M. (2013). Periodontal disease and rheumatoid arthritis: The evidence accumulates for complex pathobiologic interactions. Curr. Opin. Rheumatol..

[6] Golub L.M., Payne J., Reinhardt R., Nieman G. (2006). Can systemic diseases co-induce (not just exacerbate) periodontitis? A hypothetical “two-hit” model. J. Dent. Res..

[7] Cheng R., Wu Z., Li M., Shao M., Hu T. (2020). Interleukin-1beta is a potential therapeutic target for periodontitis: A narrative review. Int. J. Oral Sci..

[8] Souza P.P., Lerner U.H. (2013). The role of cytokines in inflammatory bone loss. Immunol. Investig..

[9] Isomaki P., Punnonen J. (1997). Pro- and anti-inflammatory cytokines in rheumatoid arthritis. Ann. Med..

[10] Papathanasiou E., Conti P., Carinci F., Lauritano D., Theoharides T.C. (2020). IL-1Β Superfamily Members and Periodontal Diseases. J. Dent. Res..

[11] Mahfooz K., Rana A., Palagati K., Suvarna A.K., Perryman C., Gaddipati S.P., Vasavada A. (2022). Anakinra in Heart Failure: A Systematic Review and Meta-Analysis of Randomized Controlled Trials. Med. Sci..

[12] Aksentijevich I., Kastner D.L. (2011). Genetics of monogenic autoinflammatory diseases: Past successes, future challenges. Nat. Rev. Rheumatol..

[13] Li Y., Ling J., Jiang Q. (2021). Inflammasomes in alveolar bone loss. Front. Immunol..

[14] Abramson S.B., Amin A. (2002). Blocking the effects of IL-1Β in rheumatoid arthritis protects bone and cartilage. Rheumatology.

[15] Neidhart M., Gay R.E., Gay S. (2000). Anti-interleukin-1 and anti-CD44 interventions producing significant inhibition of cartilage destruction in an in vitro model of cartilage invasion by rheumatoid arthritis synovial fibroblasts. Arthritis Rheum..

[16] Seckinger P., Kaufmann M.T., Dayer J.M. (1990). An interleukin 1 inhibitor affects both cell-associated interleukin 1-induced T cell proliferation and PGE2/collagenase production by human dermal fibroblasts and synovial cells. Immunobiology.

[17] Nishihara T., Ohsaki Y., Ueda N., Saito N., Mundy G.R. (1994). Mouse interleukin-1 receptor antagonist induced by *Actinobacillus actinomycetemcomitans* lipopolysaccharide blocks the effects of interleukin-1 on bone resorption and osteoclast-like cell formation. Infect. Immun..

[18] Kitazawa R., Kimble R.B., Vannice J.L., Kung V.T., Pacifici R. (1994). Interleukin-1 receptor antagonist and tumor necrosis factor binding protein decrease osteoclast formation and bone resorption in ovariectomized mice. J. Clin. Investig..

[19] Joosten L.A., Lubberts E., Helsen M.M., Saxne T., Coenen-de Roo C.J., Heinegard D., van den Berg W.B. (1999). Protection against cartilage and bone destruction by systemic interleukin-4 treatment in established murine type II collagen-induced arthritis. Arthritis Res..

[20] Bendele A., McAbee T., Sennello G., Frazier J., Chlipala E., McCabe D. (1999). Efficacy of sustained blood levels of interleukin-1 receptor antagonist in animal models of arthritis: Comparison of efficacy in animal models with human clinical data. Arthritis Rheum..

[21] Ascone G., Cao Y., Jansen I.D., Di Ceglie I., Bosch M.H.v.D., Blom A.B., van Lent P.L., Everts V., de Vries T.J. (2020). Increase in the Number of Bone Marrow Osteoclast Precursors at Different Skeletal Sites, Particularly in Long Bone and Jaw Marrow in Mice Lacking IL-1ΒRA. Int. J. Mol. Sci..

[22] Cao Y., Jansen I.D., Sprangers S., Stap J., Leenen P.J., Everts V., de Vries T.J. (2016). IL-1Βbeta differently stimulates proliferation and multinucleation of distinct mouse bone marrow osteoclast precursor subsets. J. Leukoc. Biol..

[23] Jiang Y., Genant H.K., Watt I., Cobby M., Bresnihan B., Aitchison R., McCabe D. (2000). A multicenter, double-blind, dose-ranging, randomized, placebo-controlled study of recombinant human interleukin-1 receptor antagonist in patients with rheumatoid arthritis: Radiologic progression and correlation of Genant and Larsen scores. Arthritis Rheum..

[24] Cohen S.B., Moreland L.W., Cush J.J., Greenwald M.W., Block S., Shergy W.J., Study G. (2004). A multicentre, double blind, randomised, placebo controlled trial of anakinra (Kineret), a recombinant interleukin 1 receptor antagonist, in patients with rheumatoid arthritis treated with background methotrexate. Ann. Rheum. Dis..

[25] Guo C., Yang X.G., Wang F., Ma X.Y. (2016). IL-1Βalpha induces apoptosis and inhibits the osteoblast differentiation of MC3T3-E1 cells through the JNK and p38 MAPK pathways. Int. J. Mol. Med..

[26] Bloemen V., Schoenmaker T., De Vries T.J., Everts V. (2010). Direct cell-cell contact between periodontal ligament fibroblasts and osteoclast precursors synergistically increases the expression of genes related to osteoclastogenesis. J. Cell. Physiol..

[27] Ritchlin C.T., Haas-Smith S.A., Hicks D.G., Schwarz E.M. (2003). Mechanisms of TNF-alpha- and RANKL-mediated osteoclastogenesis and bone resorption in psoriatic arthritis. J. Clin. Investig..

[28] de Vries T.J., El Bakkali I., Kamradt T., Schett G., Jansen I.D.C., D’Amelio P., Jansen I.D.C. (2019). What Are the Peripheral Blood Determinants for Increased Osteoclast Formation in the Various Inflammatory Diseases Associated with Bone Loss?. Front. Immunol..

[29] Brunetti G., Colucci S., Pignataro P., Coricciati M., Mori G., Cirulli N., Grano M. (2005). T cells support osteoclastogenesis in an in vitro model derived from human periodontitis patients. J. Periodontol..

[30] Salamanna F., Maglio M., Borsari V., Giavaresi G., Aldini N.N., Fini M. (2016). Peripheral Blood Mononuclear Cells Spontaneous Osteoclastogenesis: Mechanisms Driving the Process and Clinical Relevance in Skeletal Disease. J. Cell. Physiol..

[31] Loo-Kirana R., Gilijamse M., Hogervorst J., Schoenmaker T., de Vries T.J. (2021). Although Anatomically Micrometers Apart: Human Periodontal Ligament Cells Are Slightly More Active in Bone Remodeling Than Alveolar Bone Derived Cells. Front. Cell Dev. Biol..

[32] Prins C.M., Ceylan M., Hogervorst J.M.A., Jansen I.D.C., Schimmel I.M., Schoenmaker T., de Vries T.J. (2024). Osteogenic differentiation of periodontal ligament fibroblasts inhibits osteoclast formation. Eur. J. Cell Biol..

[33] de Vries T.J., Yousovich J., Schoenmaker T., Scheres N., Everts V. (2016). Tumor necrosis factor-alpha antagonist infliximab inhibits osteoclast formation of peripheral blood mononuclear cells but does not affect periodontal ligament fibroblast-mediated osteoclast formation. J. Periodontal Res..

[34] Oostlander A.E., Everts V., Schoenmaker T., Bravenboer N., van Vliet S.J., van Bodegraven A.A., Lips P., de Vries T.J. (2012). T cell-mediated increased osteoclast formation from peripheral blood as a mechanism for Crohn’s disease-associated bone loss. J. Cell Biochem..

[35] Zhao Y., Cai B., Zhu W., Shi J., Wang Y., Si M. (2021). IL-1Β Receptor Antagonist Protects the Osteogenesis Capability of Gingival-Derived Stem/Progenitor Cells under Inflammatory Microenvironment Induced by *Porphyromonas gingivalis* Lipopolysaccharides. Stem Cells Int..

[36] Lee Y.S., Kim Y.S., Lee S.Y., Kim G.H., Kim B.J., Lee S.H., Lee K.U., Kim G.S., Kim S.W., Koh J.M.L. (2010). AMP kinase acts as a negative regulator of RANKL in the differentiation of osteoclasts. Bone.

[37] Ruscitti P., Cipriani P., Carubbi F., Liakouli V., Zazzeroni F., Di Benedetto P., Berardicurti O., Alesse P., Giacomelli R. (2015). The role of IL-1Βbeta in the bone loss during rheumatic diseases. Mediat. Inflamm..

[38] Zamri F., de Vries T.J. (2020). Use of TNF Inhibitors in Rheumatoid Arthritis and Implications for the Periodontal Status: For the Benefit of Both?. Front. Immunol..

